# Functional Characterization of Variants in *LARP7*: Report of Three New Individuals With Alazami Syndrome and a Literature Review

**DOI:** 10.1155/humu/6490124

**Published:** 2025-06-12

**Authors:** Anastasia Ambrose, Oana Caluseriu, Saadet Mercimek-Andrews

**Affiliations:** ^1^Department of Medical Genetics, Faculty of Medicine and Dentistry, University of Alberta, Edmonton, Alberta, Canada; ^2^Women and Children's Health Research Institute, University of Alberta, Edmonton, Alberta, Canada; ^3^Neuroscience and Mental Health Institute, University of Alberta, Edmonton, Alberta, Canada; ^4^Alberta Health Services, Edmonton Zone, Alberta, Canada

**Keywords:** Alazami syndrome, congenital cardiac malformation, *LARP7*, mRNA protein expression, neurodevelopmental disorders

## Abstract

**Introduction:** Biallelic pathogenic variants in *LARP7* result in Alazami syndrome, which is characterized by global developmental delay, cognitive dysfunction, and dysmorphic features. Cardiac and skeletal phenotypes are reported in about 30% of individuals. We report three new individuals with Alazami syndrome and functional characterization of *LARP7* variants in this study.

**Materials and Methods:** We reviewed electronic patient charts. We applied the American College of Medical Genetics and Genomics and the Association for Molecular Pathology variant classification algorithms. We performed a 3D protein modeling tool for in silico prediction and functional characterization of *LARP7* variants using qPCR gene expression experiments. We reviewed the medical literature for Alazami syndrome and *LARP7*.

**Results:** We report three individuals from two unrelated families with characteristic phenotypes suggestive of Alazami syndrome. We identified a homozygous novel missense *LARP7* likely pathogenic variant (p.Asp54Val) in Family 1 and a homozygous novel pathogenic *LARP7* variant (p.Lys219Glu∗) in Family 2 using clinical exome sequencing. 3D protein modeling showed large structural changes for both variants compared to wildtype. The functional characterization showed a statistically significant difference in LARP7 expression between affected individuals and wildtype control. We report phenotypic variability within the same family that the cardiac phenotype was only present in Family 1, Case 2. There were < 60 individuals with Alazami syndrome reported to date.

**Conclusion:** We report three new individuals with Alazami syndrome and two novel variants in *LARP7*. We report the first missense *LARP7* variant associated with Alazami syndrome. We report the protein 3D structure of *LARP7* variants. We show a relationship between the p.Asp54Val *LARP7* variant and LARP7 expression levels. We think that this could be due to abnormal RNA binding of LARP7 as per the 3D protein modeling prediction tool.

## 1. Introduction

Biallelic pathogenic variants in La ribonucleoprotein domain family member 7 gene (*LARP7*) (MIM# 612026) result in intellectual disability and microcephaly that were first reported by Najmabadi et al. in 2011 [1]. The following year, Alazami et al. reported a large family with biallelic pathogenic variants in *LARP7* and called this genetic disease Alazami syndrome (MIM# 615071) [2]. Alazami syndrome is a neurodevelopmental disorder characterized by global developmental delay, intellectual disability, behavioral problems, and failure to thrive or short stature [2]. Dysmorphic features include microcephaly, malar hypoplasia, low-set ears, deep-set eyes, broad nose with flat nasal bridge or wide nasal bridge, a short philtrum, macrostomia, and full lips [2]. The skeletal phenotype is characterized by scoliosis and epiphyseal changes in the proximal phalanges [1–20]. Alazami syndrome is fully penetrant, and *LARP7* is expressed throughout the whole body with the highest expression in the brain. Variable expression of LARP7 in different tissues might result in phenotypic variability between individuals [1–20].

LARP7 protein, encoded by *LARP7*, belongs to a family of La-related proteins [21] and serves as an RNA-binding protein that binds 7SK small nuclear RNA (snRNA), acting as a central component of the 7SK ribonucleoprotein (RNP) complex. It functions as a negative regulator of transcription elongation by RNA polymerase II [22, 23]. The 7SK RNP complex binds the positive transcription elongation factor b (P-TEFb), preserving it in an inactive state within the larger 7SK RNP complex and inhibiting the phosphorylation of RNA polymerase II, which blocks transcriptional elongation [22, 23]. The 7SK RNP complex facilitates snRNA gene transcription by RNA polymerase II through its interaction with the little elongation complex [24]. LARP7 selectively binds the 3⁣′-terminal U-rich region of 7SK RNA and remains attached during activation as P-TEFb is released from the complex [21, 22]. Additionally, LARP7 promotes U6 snRNA processing, thereby regulating mRNA splicing fidelity [25]. *LARP7* is expressed throughout the whole body with the highest expression in the brain [2].

In this study, we hypothesized that two affected siblings with the *LARP7* variant of uncertain significance (VUS) had a phenotypic match of Alazami syndrome. To confirm our hypothesis, we performed functional characterization of the *LARP7* VUS. We included an individual with Alazami syndrome who was diagnosed by clinical exome sequencing as a positive control. We also applied 3D protein modeling to predict the effects of *LARP7* variants on the protein structure as a prediction tool. We report three new individuals with Alazami syndrome with two novel variants in our study. We report the first missense *LARP7* variant to be causative of Alazami syndrome. We reviewed the literature for all individuals with Alazami syndrome and compared with our individuals' phenotypes and genotypes.

## 2. Materials and Methods

The study was approved by the Institutional Research Ethics Board. The signed consent form was received as per Institutional Research Ethics Board rules (Approval Number Pro00109897). The study is also approved by the Northern Alberta Clinical Trials and Research Centre (NACTRC), and the Alberta Health Services provided operational and administrative approval (RA100656).

We reviewed electronic patient charts for the clinical history, family history, physical examination, biochemical investigations, cardiac assessments, neuroimaging, and molecular genetics and genomic investigations.

Clinical exome sequencing using patients and parents' DNA samples were performed in clinical molecular genetic laboratories according to their methods. The American College of Medical Genetics and Genomics and the Association for Molecular Pathology (ACMG/AMP) variant classification guidelines were applied for the interpretation of genetic variants [26, 27]. All variants were searched in the Genome Aggregation Database (gnomAD v4.1.0) (http://gnomad.broadinstitute.org/about) for their allele frequency in the general population [28].

We performed 3D protein modeling for the prediction of wildtype (WT) and variant protein structures for the *LARP7* variants. We retrieved protein FAST-All (FASTA) sequences from the UniProt database and input them to the AlphafoldColab2 program for 2D structure visualization and processing. We retrieved output data files from AlphafoldColab2 and input them to PyMol 2.5.5 Edu for 3D structure visualization of the protein structures. We assessed sequence coverage and confidence levels for the reliability and validity of structures. We performed all imaging of structures in accordance with the sequence alignment of variant protein structures with WT protein structures. We assessed variant protein models for predicted change in structure and amino acid interaction in relation to WT. We labelled variant residues for each variant.

We performed a literature review using *LARP7* and Alazami syndrome key words in PubMed. All references were reviewed for additional case reports in the published articles.

### 2.1. Functional Analysis of the Variants in *LARP7*

RNA was isolated from human blood from Family 1, Family 2, and WT controls (a 54-year-old East-Asian female, a 28-year-old Caucasian female, and a 32-year-old Middle Eastern female) using the RNeasy Blood RNA Kit (Qiagen, cat#762164), as per the manufacturer's instructions. The isolated RNA was reverse transcribed into cDNA using the High-Capacity cDNA Reverse Transcription Kit (Life Technology, cat# 4368814) as per the manufacturer's instructions. The synthesized cDNA was diluted at a ratio of 1:10 in ddH_2_O for use as a template for quantitative PCR (qPCR) analysis to determine LARP7 expression.

All primers and probes were synthesized by Integrated DNA Technologies Inc. (IDT). The following human *LARP7* primer sequences were used: Forward 5⁣′-CAGGTGCTTGCAGATATTGC-3⁣′; Reverse ⁣′-TTCATTTTGTTAAAAGACACAAGTAGTG-3⁣′; Probe 5⁣′-TGCATCCCCAAACCAGAAGTCCA-3⁣′. The following human GAPDH primer sequences were used: Forward 5⁣′-CCCTTCATTGACCTCAACTACA-3⁣′; Reverse 5⁣′-ATGACAAGCTTCCCGTTCTC-3⁣′; Probe 5⁣′-CAAATTCCATGGCACCGTCAAGGC-⁣′.

The real-time PCR mixture was prepared for each sample using: 4.17 *μ*L of Taqman Universal Master Mix II, with UNG (Life Technology, cat#4440012); 0.4 *μ*L of primer mixture (Forward/Reverse); 3.43 *μ*L of ddH_2_O; and 5 *μ*L of diluted cDNA. The PCR mixture was loaded onto a 384-well plate (Life Technology, cat#4309849). The expression levels were quantified using the QuantStudio 6 Pro Real-Time PCR System (Applied Biosystems) with Design & Analysis software 2.6.0. All qPCR experiments were performed in triplicates for Family 1, Family 2, and three WTs.

For each sample, technical replicates were averaged using the geometric mean to obtain a single quantification cycle (Cq) value per gene. Relative expression of LARP7 was calculated using the ΔΔCq method [29], normalizing to the geometric mean of two housekeeping genes, GAPDH and actin, in accordance with best practices for accurate and reliable normalization [30]. Statistical significance for each of the three cases and both parents from Family 1 was assessed using a one-sample *t*-test, in which each individual's normalized expression value was compared to the geometric mean of the WT group. Results were considered statistically significant at a *p* value ≤ 0.05. All calculations and qPCR visualizations were performed in R (v4.4.0).

## 3. Results

### 3.1. Family 1, Case 1

This is a 17-year-old female with global developmental delay, cognitive dysfunction, failure to thrive, and dysmorphic features who was referred to the medical genetic clinic at the age of 15 years. The pregnancy was complicated by a pre-eclampsia and intrauterine growth retardation (IUGR) in the third trimester. She was born vaginally at term. Her birth weight was < 3rd percentile. She had failure to thrive and difficulty in latching and feeding in the neonatal period, which improved with bottle feeding. At 18 months of age, she presented with a febrile seizure. She had two more febrile seizures following the first febrile seizure. She was not treated with antiseizure medications, and she did not have ongoing seizures after the three febrile seizures. She acquired age-appropriate gross and fine motor milestones in the first 1.5 years of life. Afterwards, she started showing signs of global developmental delay. She was able to walk upstairs at age 3 years. She was able to hop on both feet at age 8 years. Her first word was spoken at age 1.5 years. She was able to identify body parts at age 2 years. She spoke two-word sentences at age 3 years and three-word sentences at age 4 years. She was able to name colors at age 8 years. She began scribbling at age 2 years. She imitated vertical lines at age 5 years. She was able to feed herself at age 6 years. She could copy a circle and a cross at age 7 years. She was able to dress herself at age 9 years and brush her teeth at age 10 years. Her family history was remarkable for an affected younger sibling. Her parents were first cousins, and her mother's parents were first cousins. She was identified with behavior problems characterized by obsessive-compulsive disorder.

Her brain magnetic resonance imaging (MRI) was normal at age 12 years. She had mild to moderate hearing loss at age 12 years. Her electrocardiogram (ECG) and echocardiogram (ECHO) were normal. A chromosomal microarray was normal. Her clinical exome sequencing revealed a novel homozygous VUS in *LARP7* (c.161A>T; p.Asp54Val) (NM_016648.4). Both parents were heterozygous for this *LARP7* VUS. We summarized ACMG classification in Supporting Information 1: Table S1 and in silico predictions in Supporting Information 1: Table S2.

Currently, she is in Grade 11 in a modified program with an aide. She has moderate intellectual disability in her psychoeducational assessment, with low adaptive functioning and delayed motor, speech, and language milestones. She has a good receptive vocabulary but a limited expressive vocabulary. In her physical examination, she has a triangular long face with malar hypoplasia, widely spaced eyes, a broad nose, a flat and wide nasal bridge, a prominent and wide nasal tip, a short philtrum, full lips, macrostomia, and widely spaced teeth.

### 3.2. Family 1, Case 2

This is a 9-year-old female, the younger sister of Case 1, who was referred together with her older sister. She has global developmental delay, attention deficit disorder, cognitive dysfunction, and failure to thrive and was referred to the medical genetics clinic at the age of 7 years. The pregnancy was complicated by pre-eclampsia and IUGR. She was delivered by a planned C-section at term. Her birth weight was < 3rd percentile. She acquired age-appropriate gross and fine motor milestones in the first 1.5 years of life. Afterwards, she started showing signs of global developmental delay. She was able to walk upstairs at age 3 years. She was able to kick a ball forward at age 4 years and jump on both feet at age 4.5 years. She was able to say “Dada” or “Mama” at age 3 years. She spoke two-word phrases at age 4 years and three-word sentences at age 5.5 years. She began scribbling at age 2.5 years, imitating vertical lines at age 5 years, and brushing her teeth at age 4.5. She was able to feed herself at age 1.5 years. Her family history was remarkable for an affected older sibling, and her parents were first cousins.

Her brain MRI was normal at age 6 years of age. She had nonstenotic bicommissural aortic valve and dilated left ventricle on echocardiography at age 7 years. Her chromosomal microarray was normal. Familial variant test identified the same variant found in her sisters' clinical exome sequencing (novel homozygous VUS (c.161A>T; p.Asp54Val) in *LARP7*; NM_016648.4) (Supporting Information 1: Table S1).

Currently, she is in Grade 3 in a modified program with an aide. She has a history of failure to thrive. In her physical examination, she has a triangular long face, malar hypoplasia, prominent forehead, synophrys, widely spaced eyes, broad nose, flat and wide nasal bridge, prominent and wide nasal tip, short philtrum, full lips, macrostomia, and widely spaced teeth.

### 3.3. Family 2, Case 1

This is a 13-year-old male with global developmental delay, autism spectrum disorder, attention deficit hyperactivity disorder, anxiety, and intellectual disability who was referred to the medical genetic clinic at the age of 7 years. He was born via spontaneous vaginal delivery at term after an unremarkable pregnancy. His birth weight was at the 50th percentile. He required a 7-day neonatal intensive care unit stay and phototherapy for jaundice caused by a large cephalohematoma. He had failure to thrive at 6 months of age. He acquired age-appropriate gross and fine motor and speech/language development in the first 1 year of life. Afterwards, he started showing signs of global developmental delay. He began walking at 2 years of age. By 7 years of age, he was able to count to four, recognized words, spelled 30–50 words, and followed three-step commands. He was diagnosed with attention deficit hyperactivity disorder and anxiety at 4 years of age. At 6 years of age, he started showing sleep disturbance characterized by not being able to maintain sleep. He was also diagnosed with gastroesophageal reflux disease (GERD) at age 6 years. His psychometric assessment revealed that his development was equivalent to a 3-year-old at the chronological age of 7 years. He was diagnosed with autism spectrum disorder at 8 years of age.

He had a normal electrocardiography and brain MRI. His hearing test revealed hearing loss, and he was fitted with a hearing aid. A sleep study showed moderate sleep fragmentation and disordered sleep. An autism/intellectual disability genetic panel revealed a novel homozygous pathogenic variant (c.651_655delGAAGA; p.Lys219Glu∗; NM_016648.4) in *LARP7*. We summarized ACMG variant classification in Supporting Information 1: Table S1 and in silico predictions in Supporting Information 1: Table S2.

Currently, he is in Grade 11 in a modified program with an aide. He has global developmental delay, autism spectrum disorder, ADHD, and anxiety. He has GERD, constipation, and urinary incontinence. He has failure to thrive (weight and height < 3rd percentile). He has chronic otitis media. He can walk but is unable to run due to an in-toeing gait and toe walking. In his physical examination, he has a prominent forehead, deep-set eyes, low-set ears, a broad nose, a large mouth, widely spaced teeth, a long philtrum, and a thin upper lip. He has lordosis of the lumbar region, sacral asymmetry, and scoliosis. There was a hyperreflexia in his neurological examination.

### 3.4. Protein Modeling

**Figure 1:**
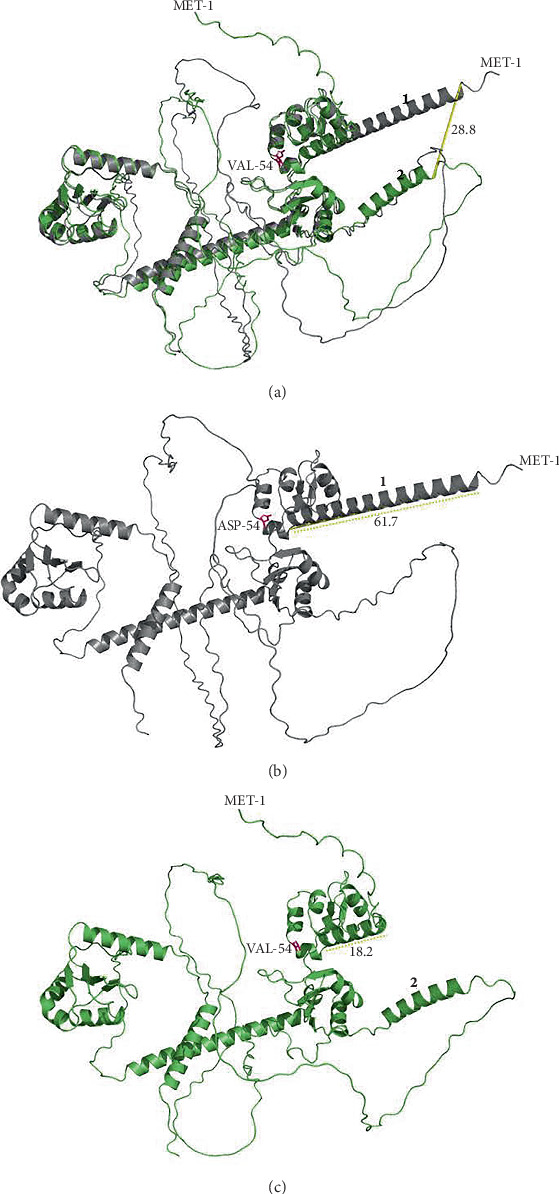
3D protein structure prediction of LARP7. Grey structures indicate wildtype protein, and green structures indicate variant protein. Variants, start and stop codons, are labelled. (a) Protein model of LARP7 c.161A>T; p.Asp54Val variant protein structure aligned with wildtype protein displays a shift in protein structure of variant compared to wildtype, including a loss of peripheral alpha helix (labelled 1) and a gain of an additional peripheral alpha helix at residue 368 (labelled 2) which measure 28.8 angstroms apart. (b) LARP7 wildtype protein structure. (c) LARP7 c.161A>T; p.Asp54Val variant protein structure.

**Figure 2:**
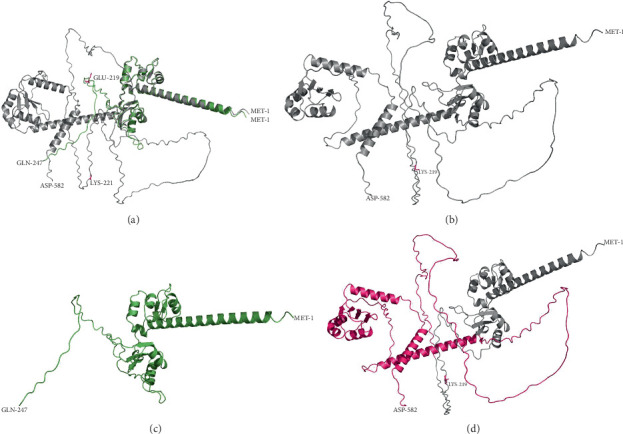
3D protein structure prediction of LARP7. Grey structures indicate wildtype protein, and green structures indicate variant protein. Variants, start and stop codons, are labelled. (a) Protein model of LARP7 c.651_655delGAAGA; p.Lys219Glu∗ variant protein structure aligned with wildtype protein displays a loss of protein due to premature termination codon and a change in conformation of the remaining protein. (b) LARP7 wildtype protein structure. (c) LARP7 c.651_655delGAAGA; p.Lys219Glu∗ variant protein structure. (d) LARP7 wildtype protein structure with residues lost due to the c.651_655delGAAGA; p.Lys219Glu∗ variant highlighted in pink.

We present the 3D protein model predictions generated using AlphaFold2 and PyMOL software for the c.161A>T; p.Asp54Val (Figure 1) and c.651_655delGAAGA; p.Lys219Glu∗ variants in *LARP7* (Figure 2). The c.161A>T; p.Asp54Val variant is predicted to induce a shift in the overall protein structure compared to WT and is predicted to lead to a partial loss of a peripheral alpha helix, truncating the first alpha helix (located at the N-terminus) at Residue 31 and formation of a new alpha helix at Residue 368 (Figure 1).

The c.651_655delGAAGA; p.Lys219Glu∗ variant is predicted to lead to a protein truncation and a loss of 335 residues compared to the wildtype protein, which is predicted to remove approximately 60% of the protein (Figure 2). The remaining protein is predicted to display an abnormal conformation and is predicted to undergo protein degradation and nonsense-mediated mRNA decay.

The validation and reliability of the outputs and the modeling results are summarized in Supporting Information 2: Figure S1, Supporting Information 3: Figure S2, and Supporting Information 4: Figure S3. All 3D protein model predictions require additional confirmation.

### 3.5. Functional Analysis

**Figure 3:**
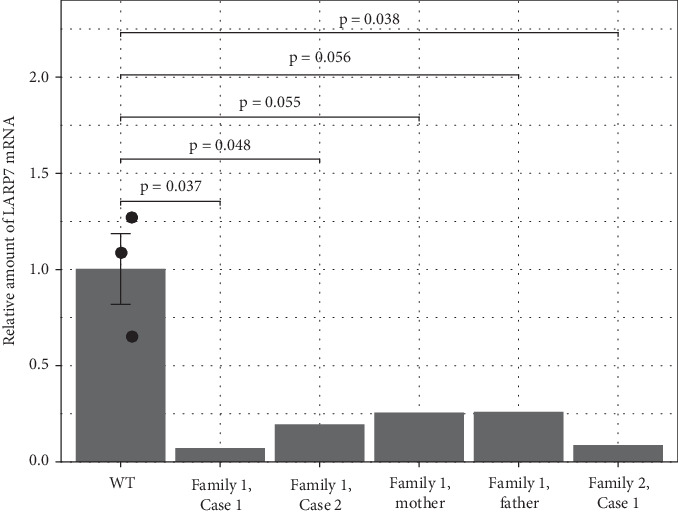
Comparative analysis of LARP7 mRNA levels in affected individuals, carriers, and controls. Results of a quantitative PCR (qPCR) analysis, showing the changes in LARP7 mRNA levels across individuals: wildtype (WT) control group (*n* = 3), an affected control (Family 2, Patient 1), the proband (Family 1, Patient 1), the affected sibling to the proband (Family 1, Patient 2), and their unaffected parents (Family 1, mother; Family 1, father). Bars represent mean relative LARP7 mRNA level ± standard error of the mean (SEM). *p* values for comparisons are included. The wildtype control serves as the baseline for comparison. LARP7 mRNA levels were normalized compared to the average of *β*-actin and GAPDH, and values for the wildtype control were set to 1.

LARP7 mRNA levels were significantly reduced in all individuals with homozygous variants in *LARP7* compared to WT control (Figure 3). All qPCR Cq values are summarized in Supporting Information 1: Table S3. Additionally, parents with heterozygous *LARP7* VUS in Family 1 showed a marked reduction in LARP7 expression compared to the WT control, although those reductions were not statistically significant.

### 3.6. Literature Review

**Figure 4:**
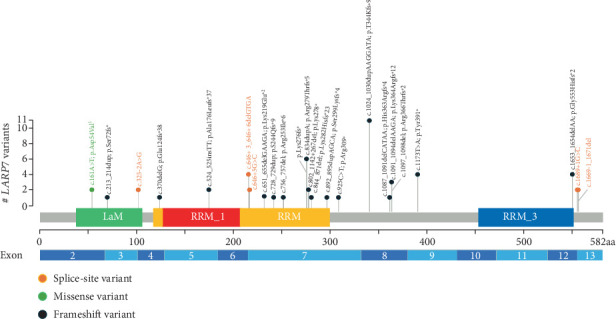
Lollipop chart of deleterious variants in *LARP7* published to date [1–19], including those in the current study indicated by superscripts 1 and 2. Two large deletions in *LARP7*, c.502_552del; p.168_184del [20] and c.641_667+25del [8], could not be depicted. Green icons indicate missense variants, orange icons indicate splicing variants, and black icons indicate frameshift variants associated with Alazami syndrome. Exons are visualized. The figure displays the protein domains of La-related protein 7, such as the La motif (LaM), RNA recognition motif (RRM), RNA Recognition Motif 1 (RRM1), and RNA Recognition Motif 2 (RRM2).

To the best of our knowledge, less than 60 individuals with pathogenic/likely pathogenic (P/LP) variants in *LARP7* have been reported [1–20]. We summarized all previously reported individuals in Supporting Information 1: Table S4. We depicted the location of all variants (including previously reported and current individuals' variants) in Figure 4. All individuals had neurodevelopmental phenotype (*n* = 51) including global developmental delay, cognitive dysfunction, psychiatric problems or behavioral problems, and dysmorphic features. More than three-quarter of the individuals had growth problems ranging from IUGR to short stature. About one-third of individuals has skeletal and cardiac phenotypes. Renal and gastrointestinal phenotypes were rare. There have been 24 different *LARP7* variants associated with Alazami syndrome including frameshift (*n* = 17), splicing (*n* = 5), and large deletion (*n* = 2).

## 4. Discussion

We report three new individuals with Alazami syndrome from two unrelated families and functional characterization of a VUS supporting the genetic diagnosis of Alazami syndrome in Family 1. We report two novel variants and the first missense variant in *LARP7.* The conformational changes in 3D protein structure and reduced LARP7 expression on qPCR led to the reclassification of the VUS to likely pathogenic. Our study emphasizes the importance of functional characterization for the VUS reclassification. As we have not performed genome sequencing, we could not rule out any other type of variants, which might have resulted in low expression of LARP7. However, we think that we were able to show a relationship between the p.Asp54Val *LARP7* variant and the LARP7 expression levels in blood.

Hearing loss, recurrent otitis media or middle ear infection, GERD, and sleep disturbances were reported in less than 10 individuals with Alazami syndrome in the medical literature. Interestingly, individuals in our study had one of these phenotypes (Supporting Information 1: Table S4) including Family 1, Case 1 with hearing loss and Family 2, Case 1 with recurrent otitis media, GERD, and sleep disorder.

Skeletal phenotypes ranged from contractures, scoliosis, advanced bone age, and calvarium changes to skeletal abnormalities in x-ray (e.g., increased cortical thickness, high vertebrae with small intervertebral disks). Interestingly, Family 2, Case 1 had lordosis of the lumbar region and sacral asymmetry. Cardiac phenotype includes atrial septal defect, pulmonary stenosis, ventricular septal defect, increased thickness of the interventricular septum, and left ventricular noncompaction in about one-third of the individuals with Alazami syndrome reported in the medical literature. Family 1, Case 2 had nonstenotic bicommissural aortic valve and dilated left ventricle in the echocardiography. As we have not found this cardiac phenotype in any of the individuals with Alazami syndrome reported previously, this might be a novel cardiac phenotype in echocardiography in Family 1, Case 2. Seizures have been reported in about 10% of the individuals with Alazami syndrome in the medical literature. Family 1, Case 1 presented with febrile seizures in our study.

Previous studies have reported phenotypic variability between siblings [1, 2, 4–7, 10, 11]. Imbert-Bouteille et al. reported keratoconus and atrial septal defect in one sibling and seizures in the other [6]. Hosseini et al. described seizures and an unbalanced gait in one sibling only [11]. Elmas et al. noted variability including pulmonary stenosis, diabetes mellitus, and retinal abnormalities in one sibling, while the other exhibited seizures and less severe systemic involvement [10]. We report two siblings with Alazami syndrome in Family 1 that display phenotypic variability. Both siblings have global developmental delay, cognitive dysfunction, failure to thrive, IUGR, and dysmorphic features. Case 1 has a history of febrile seizures, obsessive-compulsive behavior, hearing loss, and moderate intellectual disability, while Case 2 has attention deficit disorder and congenital cardiac malformation in echocardiography. Interestingly, LARP7 expression was different between these siblings. The phenotypic variability within the same family might be due to different expression levels of LARP7 in heart tissue in Case 2. As we do not have expression studies in different tissues, we will not be certain to explain phenotypic variability between the two siblings in our study.

Functional characterization of *LARP7* variants were reported in few studies previously. Functional characterization of the p.T344Kfs∗9 *LARP7* variant using immunoblotting revealed a total absence of LARP7, and real-time PCR showed reduced LARP7 and 7SK RNA levels in lymphoblasts and fibroblasts [2]. Holohan et al. analyzed telomere length in individuals' blood DNA using terminal restriction fragment (TRF) assays, revealing significantly shorter telomeres compared to healthy controls [3]. They performed flow-FISH analysis and found decreases in full-length functional mRNA and the nonfunctional beta isoform, implicating LARP7 in telomerase mRNA splicing [3]. In our study, we performed a qPCR gene expression experiment and measured the relative expression levels of LARP7 in three individuals with Alazami syndrome compared to WT control. Interestingly, the p.Asp54Val variant showed similarly decreased LARP7 expression compared to the p.Lys219Glu∗ variant. This suggests that the missense variant impairs LARP7 expression to a level similar to a loss of function variant. Additionally, the expression of LARP7 was low in both parents (in mother 25.6% and in father 26% of normal) in Family 1. As the mother and father were asymptomatic, we did not perform echocardiography in both. As the parents and maternal grandparents were cousins, there might be some other epigenetic contributions to the LARP7 expression in both parents that we are not aware of. Tissue-specific regulation, differential expression patterns, transcriptional regulatory effects, unequal allelic expression, and compensatory mechanisms could contribute to low LARP7 expression in blood in both parents. To the best of our knowledge, LARP7 expression was not reported in parents previously to compare with our study results. LARP7 has a La motif and three RNA recognition motifs (RRM, RRM1, and RRM3) [22]. The La motif and RRM1 are involved in the interaction of La with the 3⁣′ end of nascent RNA polymerase III transcripts [21]. LARP7 is required for the stability of 7SK RNA [21, 23]. The protein is also involved in the regulation of P-TEFb, which is a key factor in the transcription of cellular genes [21, 23]. The p.Asp54Val *LARP7* variant is in Exon 2 and affects the La motif of LARP7 (Figure 4). It replaces the polar, negatively charged aspartic acid with a nonpolar, neutral valine. 3D protein modeling of the p.Asp54Val *LARP7* variant induces truncation of the first alpha helix and the formation of a new alpha helix at Residue 368. As the La motif in LARP7 is required for its interaction with nascent RNAs, alteration of the La motif structure may affect LARP7 ability to bind RNA substrates, impairing the regulation of RNA polymerase III activity due to its location in the La motif domain. There are two *LARP7* variants, one is a small duplication (cause premature termination) and one is a splice site (predicted to disrupt the conserved acceptor splice site of Exon 6) (Figure 4) in the La motif domain [7, 14]. There are several potential mechanisms by which a missense variant in *LARP7* could result in low mRNA levels in Family 1 [31–33] including: (1) Missense variants can disrupt splicing by altering exonic splicing enhancers or silencers, potentially leading to exon skipping, intron retention, or cryptic splice site activation—events that can introduce premature termination codons and trigger nonsense-mediated decay (NMD), ultimately reducing mRNA levels [31, 32]. (2) Missense variants could activate a cryptic polyadenylation site, resulting in a truncated and unstable mRNA transcript, although this is less common for coding-region variants [32]. (3) Changes in mRNA structure introduced by missense variants may impact the binding of RNA-binding proteins that regulate mRNA stability, either promoting degradation or preventing stabilization [32]. (4) Missense variants may indirectly affect transcription, such as variations in transcription factor binding sites affecting gene expression [32]. Given the established role of LARP7 in RNA polymerase II transcriptional regulation and 7SK snRNP stability, disruptions at the transcript level could have broad downstream effects. The c.651_655delGAAGA; p.Lys219Glu∗ frameshift variant, in the RRM domain, leads to premature protein truncation and a loss of 335 residues compared to the WT protein. The remaining protein is predicted to undergo protein degradation and nonsense-mediated mRNA decay, which likely leads to loss of function of LARP7. RRM domains are involved in the binding of single stranded RNA and affect sequence and shape dependent RNA recognition as well as protein–protein interaction. We think that protein 3D modeling gives us valuable information for structural changes of proteins caused by the variants identified to help us understand their pathogenicity in the absence of further functional characterization.

In conclusion, we report three new individuals with Alazami syndrome with two novel variants in our study. We report the first missense *LARP7* variant to be causative of Alazami syndrome. We report for the first time protein 3D structure of variants in *LARP7*. We were able to show a relationship between the p.Asp54Val *LARP7* variant and LARP7 expression levels. We think that this could be due to abnormal RNA binding of LARP7 as per 3D protein modeling prediction tool.

## Data Availability

All data generated or analyzed during this study are included in this published article and its supporting information files.

## Supporting Information

Supporting Information 1 Table S1: ACMG/AMP variant classification of identified variants is summarized in Table S1. Abbreviations: del = deletion, NR = not reported, VUS = variant of uncertain significance. Table S2: In silico predictions of the identified variants are summarized in Table S2. Abbreviations: NR = not reported. Table S3: qPCR results of all individuals are included in Table S3. Abbreviations: Cq = quantification cycle, UD = undetermined, WT = wildtype. Table S4: All previously reported cases with Alazami syndrome are summarized in Table S4.

Supporting Information 2 Figure S1: AlphaFold predictions for sequence coverage, pLDDT, and PAE, assessing the reliability and validity of wildtype protein models. (A) pLDDT scores per residue, indicating local confidence in the structural prediction. (B) Sequence coverage of wildtype protein models. (C) PAE values, representing predicted alignment errors and domain positioning uncertainty. Abbreviations: PAE = predicted aligned error, pLDDT = predicted local distance difference test.

Supporting Information 3 Figure S2: AlphaFold predictions for sequence coverage, pLDDT, and PAE, assessing the reliability and validity of the c.161A>T; p.Asp54Val variant protein models. (A) pLDDT scores per residue, indicating local confidence in the structural prediction. (B) Sequence coverage of the c.161A>T; p.Asp54Val variant protein models. (C) PAE values, representing predicted alignment errors and domain positioning uncertainty of the c.161A>T; p.Asp54Val variant protein models. Abbreviations: PAE = predicted aligned error, pLDDT = predicted local distance difference test.

Supporting Information 4 Figure S3: AlphaFold predictions for sequence coverage, pLDDT, and PAE, assessing the reliability and validity of c.651_655delGAAGA; p.Lys219Glu∗ variant protein models. (A) pLDDT scores per residue, indicating local confidence in the structural prediction. (B) Sequence coverage of c.651_655delGAAGA; p.Lys219Glu∗ variant protein models. (C) PAE values, representing predicted alignment errors and domain positioning uncertainty of c.651_655delGAAGA; p.Lys219Glu∗ variant protein models. Abbreviations: PAE = predicted aligned error, pLDDT = predicted local distance difference test.
