# Supplementary material for: Functional Characterization of Variants in LARP7: Report of Three New Individuals With Alazami Syndrome and a Literature Review
Source: Hum Mutat. 2025 Jun 12;2025:6490124. doi: 10.1155/humu/6490124 (PMC12178771; doi:10.1155/humu/6490124)
Supplement: Supporting Information 1 — Table S1: ACMG/AMP variant classification of identified variants is summarized in Table S1. Abbreviations: del = deletion, NR = not reported, VUS = variant of uncertain significance. Table S2: In silico predictions of the identified variants are summarized in Table S2. Abbreviations: NR = not reported. Table S3: qPCR results of all individuals are included in Table S3. Abbreviations: Cq = quantification cycle, UD = undetermined, WT = wildtype. Table S4: All previously reported cases with Alazami syndrome are summarized in Table S4. [file 6490124.f1.zip › Supplemental_Tables_Rev2_Clean.pdf]

Supplemental Tables

Title: Functional characterization of variants in *LARP7*: Report of three new individuals with Alazami syndrome and a literature review

Anastasia Ambrose[1], Oana Caluseriu[1,3], Saadet Mercimek-Andrews*[1,2,3,4]

[1] Department of Medical Genetics, Faculty of Medicine and Dentistry, University of Alberta, Edmonton, Alberta, Canada

[2] Neuroscience and Mental Health Institute, University of Alberta, Edmonton, Alberta, Canada

[3] Women and Children's Health Research Institute, University of Alberta, Edmonton, Alberta, Canada

[4] Alberta Health Services, Edmonton Zone, Alberta, Canada

***Corresponding author:**

Saadet Mercimek-Andrews, MD, PhD, FCCMG

Professor, Department of Medical Genetics, University of Alberta

8-39 Medical Sciences Building

8613 114 Street

Edmonton, AB T6G 2H7

Phone: 780.492.0874

Fax: 780.492.1998

E-mail: saadet@ualberta.ca

Supplemental Table 1. ACMG/AMP variant classification of identified variants are summarized in Supplemental Table 1.

**Gene NM#/Inheritance Pattern**	**Variant (Zygosity/Inheritance)**	**SIFT**	**MuTaster**	**Polyphen2 (HumVar)**	**Conservation in Species**	**gnomAD allele frequency**	**Variant Type**	**Variant classification**
LARP7 (NM_016648.4)/ AR	c.161A>T; p. Asp54Val (homozygous /maternal, paternal)	Deleterious	Disease causing	Probably damaging	8 out of 8	0	Missense	Likely pathogenic (PM2_supporting, PP3, PS3_supporting, PM3_supporting, PP1_moderate)
LARP7 (NM_016648.4)/ AR	c.651_655delGAAGA; p.Lys219Glu*	NR	NR	NR	NR	0	Frameshift	Pathogenic (PVS1, PM2_supporting,

	(homozygous / maternal, paternal)							PM3_supporting, PP5)

Abbreviations: del= deletion; NR= not reported; VUS= variant of uncertain significance

Supplemental Table 2. In-silico predictions of the identified variants are summarized in Supplemental Table 2.

**In-silico tools**	**Predictions**	**Score**
***LARP7* c.161A>T; p. Asp54Val**		
MUT assessor	Hi	3.97
SIFT	Deleterious	0
PolyPhen-2	Damaging	0.97
MutTaster	Disease causing	1
FATHMM	Uncertain	-0.51
DANN	Deleterious	0.99
MetaLR	Deleterious (low)	0.67
Revel	Deleterious (moderate)	0.89
PrimateAI	Uncertain	0.77
gnomAD	0	NA
Conservation in species	8/8	NA
LARP7* c.651_655delGAAGA, p.Lys219Glu		
MUT assessor	NR	NR
SIFT	NR	NR
PolyPhen-2	NR	NR
MutTaster	NR	NR
FATHMM	NR	NR
DANN	NR	NR
MetaLR	NR	NR

Revel	NR	NR
PrimateAI	NR	NR
gnomAD	0	NR
Conservation in species	NR	NR

Abbreviations: NR= not reported

Supplemental Table 3. qPCR results of all individuals are included in Supplemental Table 3.

Individuals	LARP7_Cq	Actin_Cq	GAPDH_Cq
Family 1, Case 1	26.18	19.88	22.57
Family 1, Case 1	26.10	18.99	22.98
Family 1, Case 1	26.26	18.69	22.58
Family 1, Case 2	24.22	18.24	22.11
Family 1, Case 2	24.35	18.40	22.68
Family 1, Case 2	23.45	17.98	22.08
Family 1, Mother	24.17	18.83	22.38
Family 1, Mother	23.74	18.61	21.22
Family 1, Mother	23.49	18.78	22.61
Family 1, Father	23.84	18.95	21.91
Family 1, Father	23.17	18.76	20.79
Family 1, Father	23.27	18.36	21.41
Family 2, Case 1 (positive control)	27.45	21.12	23.17
Family 2, Case 1 (positive control)	26.66	20.53	23.73
Family 2, Case 1 (positive control)	26.94	20.40	23.23
Wildtype _1	24.11	22.09	UD
Wildtype _1	24.23	21.52	24.22
Wildtype _1	24.03	20.87	23.91
Wildtype _2	23.54	UD	UD
Wildtype _2	24.39	UD	23.81
Wildtype _2	23.43	19.70	23.83

Wildtype _3	24.46	22.60	23.91
Wildtype _3	24.65	23.58	23.79
Wildtype_3	24.20	23.90	21.98

Abbreviations: Cq= quantification cycle; UD= undetermined; WT=wildtype

Supplemental Table 4. All previously reported cases with Alazami syndrome are summarized in Supplemental Table 4

STROBE Statement—checklist of items that should be included in reports of observational studies

	Item No.	Recommendation	Page No.	Relevant text from manuscript
**Title and abstract**	1	(*a*) Indicate the study's design with a commonly used term in the title or the abstract	1, 2	Lines 22-45
		(*b*) Provide in the abstract an informative and balanced summary of what was done and what was found	1, 2	Lines 22-45
**Introduction**				
Background/rationale	2	Explain the scientific background and rationale for the investigation being reported	3,4	Lines 51-74
Objectives	3	State specific objectives, including any prespecified hypotheses	4	Lines 73-74
**Methods**				
Study design	4	Present key elements of study design early in the paper	4,5,6	Lines 76-121
Setting	5	Describe the setting, locations, and relevant dates, including periods of recruitment, exposure, follow-up, and data collection	4,5,6	Lines 76-121
Participants	6	(*a*) *Cohort study*—Give the eligibility criteria, and the sources and methods of selection of participants. Describe methods of follow-up<br>*Case-control study*—Give the eligibility criteria, and the sources and methods of case ascertainment and control selection. Give the rationale for the choice of cases and controls<br>*Cross-sectional study*—Give the eligibility criteria, and the sources and methods of selection of participants	4,5,6	Lines 76-121
		(*b*) *Cohort study*—For matched studies, give matching criteria and number of exposed and unexposed<br>*Case-control study*—For matched studies, give matching criteria and the number of controls per case		
Variables	7	Clearly define all outcomes, exposures, predictors, potential confounders, and effect modifiers. Give diagnostic criteria, if applicable	4,5,6	Lines 76-121
Data sources/ measurement	8*	For each variable of interest, give sources of data and details of methods of assessment (measurement). Describe comparability of assessment methods if there is more than one group	4,5,6	Lines 76-121
Bias	9	Describe any efforts to address potential sources of bias	NA	
Study size	10	Explain how the study size was arrived at	4	76-82

Continued on next page

Quantitative variables	11	Explain how quantitative variables were handled in the analyses. If applicable, describe which groupings were chosen and why	5,6	101-121
Statistical methods	12	(*a*) Describe all statistical methods, including those used to control for confounding	6	119-121
		(*b*) Describe any methods used to examine subgroups and interactions	6	119-121
		(*c*) Explain how missing data were addressed	NA	
		(*d*) *Cohort study*—If applicable, explain how loss to follow-up was addressed<br>*Case-control study*—If applicable, explain how matching of cases and controls was addressed<br>*Cross-sectional study*—If applicable, describe analytical methods taking account of sampling strategy	NA	
		(*e*) Describe any sensitivity analyses	6,19	
**Results**				
Participants	13*	(a) Report numbers of individuals at each stage of study—eg numbers potentially eligible, examined for eligibility, confirmed eligible, included in the study, completing follow-up, and analysed	6-9	122-203
		(b) Give reasons for non-participation at each stage	NA	
		(c) Consider use of a flow diagram	NA	
Descriptive data	14*	(a) Give characteristics of study participants (eg demographic, clinical, social) and information on exposures and potential confounders	6-9	122-203
		(b) Indicate number of participants with missing data for each variable of interest	NA	
		(c) *Cohort study*—Summarise follow-up time (eg, average and total amount)	6-9	122-203
Outcome data	15*	*Cohort study*—Report numbers of outcome events or summary measures over time	6-10	122-221
		Case-control study—Report numbers in each exposure category, or summary measures of exposure	6-10	122-221
		Cross-sectional study—Report numbers of outcome events or summary measures	6-10	122-221
Main results	16	(*a*) Give unadjusted estimates and, if applicable, confounder-adjusted estimates and their precision (eg, 95% confidence interval). Make clear which confounders were adjusted for and why they were included	10, 24	215-221, Supplemental Table 3
		(*b*) Report category boundaries when continuous variables were categorized	NA	
		(*c*) If relevant, consider translating estimates of relative risk into absolute risk for a meaningful time period	NA	

Continued on next page

Other analyses	17	Report other analyses done—eg analyses of subgroups and interactions, and sensitivity analyses	10, 24	215-221, Supplemental Table 3
**Discussion**				
Key results	18	Summarise key results with reference to study objectives	11-14	234-301
Limitations	19	Discuss limitations of the study, taking into account sources of potential bias or imprecision. Discuss both direction and magnitude of any potential bias	NA	
Interpretation	20	Give a cautious overall interpretation of results considering objectives, limitations, multiplicity of analyses, results from similar studies, and other relevant evidence	11-14	234-301
Generalisability	21	Discuss the generalisability (external validity) of the study results	13	227-301
**Other information**				
Funding	22	Give the source of funding and the role of the funders for the present study and, if applicable, for the original study on which the present article is based	15	308-309

*Give information separately for cases and controls in case-control studies and, if applicable, for exposed and unexposed groups in cohort and cross-sectional studies.

Note: An Explanation and Elaboration article discusses each checklist item and gives methodological background and published examples of transparent reporting. The STROBE checklist is best used in conjunction with this article (freely available on the Web sites of PLoS Medicine at http://www.plosmedicine.org/, Annals of Internal Medicine at http://www.annals.org/, and Epidemiology at http://www.epidem.com/). Information on the STROBE Initiative is available at www.strobe-statement.org.
