# Supplementary figures and images for: Functional Characterization of Variants in LARP7: Report of Three New Individuals With Alazami Syndrome and a Literature Review
Source: Hum Mutat. 2025 Jun 12;2025:6490124. doi: 10.1155/humu/6490124 (PMC12178771; doi:10.1155/humu/6490124)

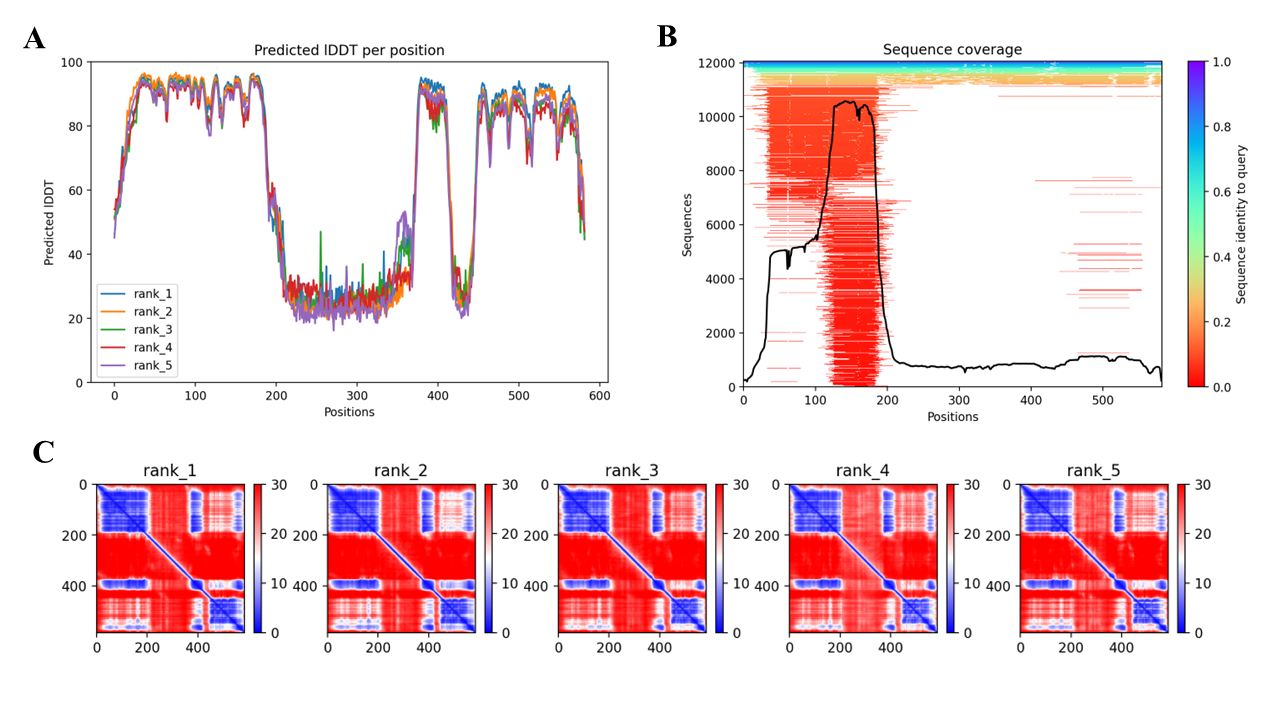

Supplement: Supporting Information 2 — Figure S1: AlphaFold predictions for sequence coverage, pLDDT, and PAE, assessing the reliability and validity of wildtype protein models. (A) pLDDT scores per residue, indicating local confidence in the structural prediction. (B) Sequence coverage of wildtype protein models. (C) PAE values, representing predicted alignment errors and domain positioning uncertainty. Abbreviations: PAE = predicted aligned error, pLDDT = predicted local distance difference test. [file 6490124.f2.tif]

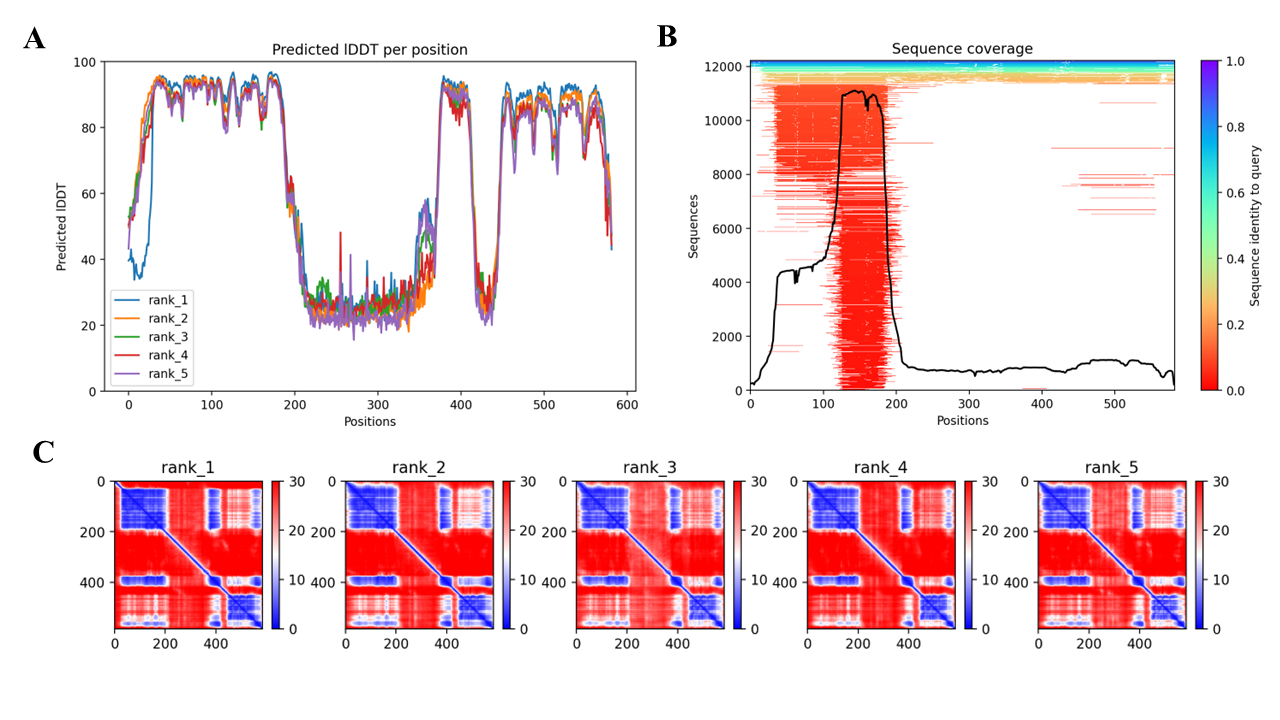

Supplement: Supporting Information 3 — Figure S2: AlphaFold predictions for sequence coverage, pLDDT, and PAE, assessing the reliability and validity of the c.161A>T; p.Asp54Val variant protein models. (A) pLDDT scores per residue, indicating local confidence in the structural prediction. (B) Sequence coverage of the c.161A>T; p.Asp54Val variant protein models. (C) PAE values, representing predicted alignment errors and domain positioning uncertainty of the c.161A>T; p.Asp54Val variant protein models. Abbreviations: PAE = predicted aligned error, pLDDT = predicted local distance difference test. [file 6490124.f3.tif]

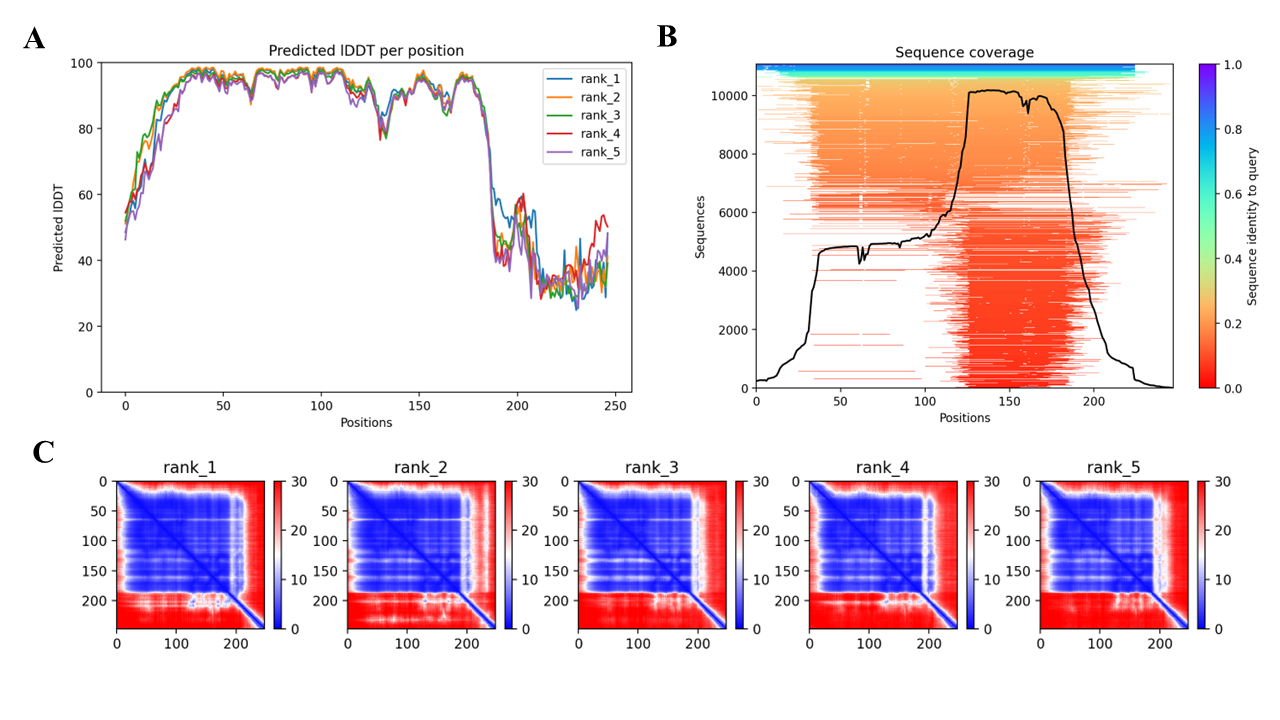

Supplement: Supporting Information 4 — Figure S3: AlphaFold predictions for sequence coverage, pLDDT, and PAE, assessing the reliability and validity of c.651_655delGAAGA; p.Lys219Glu∗ variant protein models. (A) pLDDT scores per residue, indicating local confidence in the structural prediction. (B) Sequence coverage of c.651_655delGAAGA; p.Lys219Glu∗ variant protein models. (C) PAE values, representing predicted alignment errors and domain positioning uncertainty of c.651_655delGAAGA; p.Lys219Glu∗ variant protein models. Abbreviations: PAE = predicted aligned error, pLDDT = predicted local distance difference test. [file 6490124.f4.tif]
